# Stochastic bursting in networks of excitable units with delayed coupling

**DOI:** 10.1007/s00422-021-00883-9

**Published:** 2021-06-28

**Authors:** Chunming Zheng, Arkady Pikovsky

**Affiliations:** 1 Institute for Physics and Astronomy, University of Potsdam, Karl-Liebknecht-Strasse 24/25, 14476 Potsdam-Golm, Germany; 2 Department of Control Theory, Nizhny Novgorod State University, Gagarin Avenue 23, Nizhny Novgorod, Russia 606950

**Keywords:** Excitable system, Noise-induced spikes, Time-delayed coupling, Point process

## Abstract

We investigate the phenomenon of *stochastic bursting* in a noisy excitable unit with multiple weak delay feedbacks, by virtue of a directed tree lattice model. We find statistical properties of the appearing sequence of spikes and expressions for the power spectral density. This simple model is extended to a network of three units with delayed coupling of a star type. We find the power spectral density of each unit and the cross-spectral density between any two units. The basic assumptions behind the analytical approach are the separation of timescales, allowing for a description of the spike train as a point process, and weakness of coupling, allowing for a representation of the action of overlapped spikes via the sum of the one-spike excitation probabilities.

## Introduction

Bursting, which plays an important role in neuronal communication and synchronization, refers to a dynamical state where a neuron repeatedly fires a relatively regular finite sequence spikes; bursts are separated by epochs where the neuron is in a resting state (Izhikevich 2000; Coombes and Bressloff 2005). Bursting is observed in neurons of different types, such as in neocortex (Connors and Gutnick 1990), hippocampus (Dzhala and Staley 2004; Su et al. 2001) and cerebellum (Womack and Khodakhah 2002); see the classification in (Izhikevich 2006b). In many situations, bursting is an intrinsic property of a neuron, following from the particular properties of its native dynamics. Correspondingly, the theory of such bursting is based on the bifurcation theory of dynamical systems (Izhikevich 2000; Rinzel 1987; Channell et al. 2007).

In our previous works (Zheng and Pikovsky 2018, 2019), we have demonstrated that a coherent spike pattern, which we call *stochastic bursting*, can appear in simple excitable units due to the combined effect of time-delayed feedback and noise. Noise plays a twofold role in the stochastic bursting. On the one hand, it leads to spontaneous appearance of quite rare statistically independent spikes. On the other hand, when a delayed feedback pulse enters like an excitatory under-threshold kick (i.e., a weak kick that itself without noise does not produce a new spike), noise results in an increased probability to create a new induced spike. Thus, a spontaneous spike (the leader) can be followed by a sequence of induced spikes (the followers) separated approximately by the delay time interval. Because the creation of a follower is a random event due to noise, the overall burst has a random number of spikes. We described stochastic bursting statistically in the case of a single excitable unit (Zheng and Pikovsky 2018) and for networks of unidirectionally delay-coupled units (Zheng and Pikovsky 2019). What these two cases have in common, is that any two delay-induced kicks do not overlap. This allowed for a full statistical description of the bursting as a point process, where the only parameters are the spontaneous rate of excitation and the probability to excite a follower. The point process model is an idealization based on the timescale separation: It is assumed that the characteristic duration of a spike is much less than other characteristic times in the system, the delay time and the characteristic time interval between the pulses (which depends on the spontaneous rate and the probability of induced spikes).

However, it becomes more challenging when neurons have multiple feedback or more complex coupling topology, where two or more delay-induced spikes could overlap. Such an overlap of incoming spikes in a purely deterministic setup let E. Izhikevich to introduce the concept of polychronization (Izhikevich 2006a). Specific polychronous patterns are determined by combinations of delay time. They appear if, for a reliable excitation, superposition of delay-induced kicks is favorable. Here, below we treat the effect of overlap of incoming kicks in a stochastic setting, assuming that the probability to induce a spike increases if two kicks overlap.

Similar to the previously studied cases of stochastic bursting (and generally for systems with noise and delay), the process under study is non-Markovian, which prevents using the powerful Fokker–Planck formalism to obtain analytic results. Thus, we adopt the point-process representation as a good option to describe the statistics of the spike train in each neuron and between different neurons, if timescale separation holds.

In the present paper, we extend the theory of stochastic bursting to the case where incoming delayed pulses can overlap. Here, generally, one needs to define the probability to induce a spike by two incoming pulses. We restrict our attention below to the case of weak coupling, where this probability can be represented through one-pulse probabilities. This allows for an analytical treatment, which results in explicit expressions for the power spectrum of the spike trains. First, in Sect. 2, we investigate the stochastic bursting phenomenon in a noisy excitable unit with multiple delay feedbacks. Then, in Sect. 3, we extend the theory to a network of three delay-coupled units, with a star-type coupling.

**Fig. 1:**
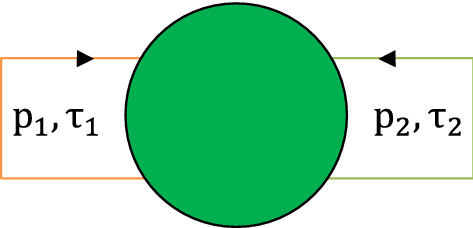
Schematic description of a noisy excitable unit with two feedback loops

## One excitable unit with multiple delayed feedbacks

The main goal of this paper is to extend the theory of stochastic bursting to the situations where an overlap of delayed feedback pulses is possible. The simplest case is one unit with two delayed feedbacks, as shown in Fig. 1. (In one unit with one delay, the incoming pulses cannot overlap.) As we see in Sect. 3, the hub unit (unit 2 in Fig. 6) in a star-type network possesses two or several feedback loops with different delays and is thus similar to the simplest setup of this section. As a model, we consider a scalar equation on a circle $0\le \theta <2\pi$, which is a prototypical example of excitability:

1 $$\begin{aligned} \begin{aligned} {\dot{\theta }}&=a+\cos \theta +\epsilon _1(a+\cos \theta (t-{\hat{\tau }}_1))\\&\quad +\epsilon _2(a+\cos \theta (t-{\hat{\tau }}_2))\\&\quad +\xi (t). \end{aligned} \end{aligned}$$

**Fig. 2:**
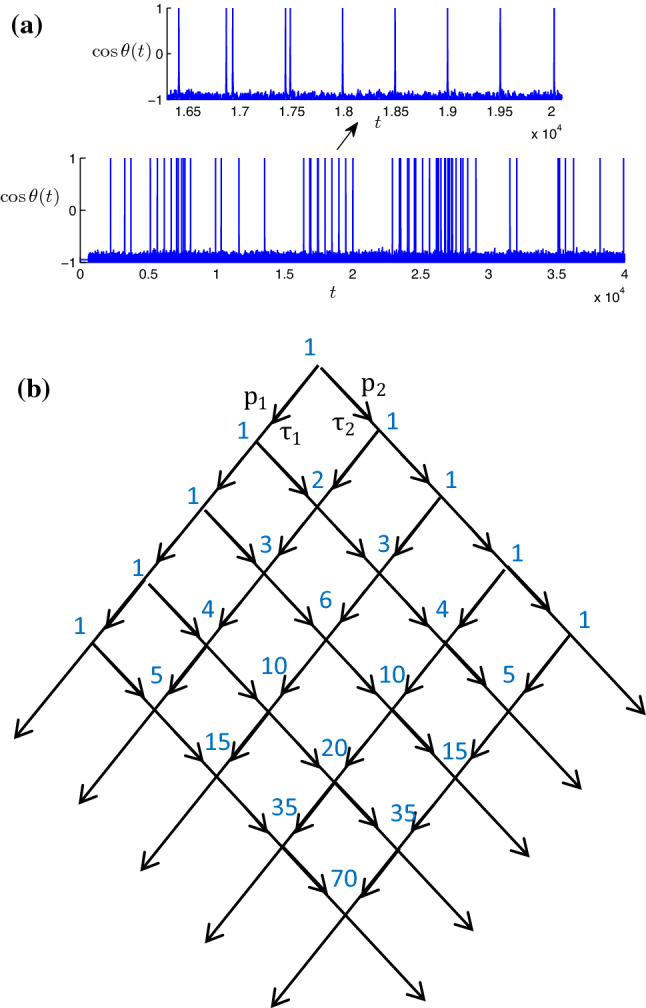
Spike train (**a**) of a neuron (1) with two feedback signals, with values of parameters chosen as $a=0.95, D=0.005, \epsilon _1=0.12, {\hat{\tau }}_1=500, \epsilon _2=0.1$ and ${\hat{\tau }}_2=600$. Zooming in a burst shows many spikes with interspike interval of around 500 and 600. (**b**) Representation in directed tree lattice, where the cross-position of the lattice, i.e., $k\tau _1+l\tau _2$ with *k*, *l* being non-negative integers, represents where there is potentially a delay-induced spike with some probability $P(k\tau _1+l\tau _2)$. The blue numbers near the intersection $k\tau _1+l\tau _2$ are the number of paths to go to that point, i.e., $$\left( {\begin{array}{c}k+l\\ k\end{array}}\right) $$ in Eq. (7)

Here, parameter $a\lesssim 1$ defines the excitability level and parameters $\epsilon _1$ and $\epsilon _2$ are coupling strengths for the two delayed feedbacks with delay times ${\hat{\tau }}_1$ and ${\hat{\tau }}_2$, respectively. The system is driven by a Gaussian white noise $\xi (t)$ with intensity *D*, i.e., $\langle \xi (t)\rangle =0$, $\langle \xi (t)\xi (t')\rangle =2D\delta (t-t')$. By direct simulation of the Langevin equation (1) using Euler–Maruyama scheme with time step ${\varDelta }t=0.01$, we observe rather coherent spiking which we call stochastic bursting in the spike train, as shown in Fig. 2a.

Without feedback (i.e., for $\epsilon _1=\epsilon _2=0$), the model is equivalent to the well-known models of active rotators (Shinomoto and Kuramoto 1986) and to the popular theta model (Ermentrout and Kopell 1986). For $a<1$, there are two steady states, one stable and one unstable. Noise drives the state of the system beyond the unstable steady state, and a rotation around the circle back to the stable state occurs; this rotation is indicated as a spike. To find statistics of the spikes, one formulates the Fokker–Planck equation for the evolution of the probability density of a noisy unit obeying Eq. (1) as follows:

2 $$\begin{aligned} \frac{\partial {\hat{P}}(\theta ,t)}{\partial t}=-\frac{\partial }{\partial \theta }\left[ (a+\cos \theta ) {\hat{P}}(\theta ,t)\right] +D\frac{\partial ^{2} {\hat{P}}(\theta ,t)}{\partial \theta ^{2}}. \end{aligned}$$

The stationary solution of (2) is

3 $$\begin{aligned} {\hat{P}}_{st}(\theta )=C\int _{\theta }^{\theta +2\pi }\frac{\mathrm{d}\psi }{D} e^{-\int _{\theta }^{\psi }\frac{a+\cos \varphi }{D}\mathrm{d}\varphi }. \end{aligned}$$

Here, *C* is a normalization constant. The probability current across threshold yields the rate of spontaneous spike excitations:

4 $$\begin{aligned} \lambda =C\left( 1-e^{-\int _{0}^{2\pi }\frac{a+\cos \theta }{D}\mathrm{d}\theta }\right) . \end{aligned}$$

Below we will assume this rate to be small, i.e., the characteristic time interval between the noise-excited spikes is much larger than the duration of the spikes. Below we will also assume that this separation of times is valid for characteristic times of the delayed feedback: The delay times ${\hat{\tau }}_1$, ${\hat{\tau }}_2$ and their difference $|{\hat{\tau }}_1-{\hat{\tau }}_2|$ are much larger than the duration of a pulse.

We now qualitatively describe the phenomenon of stochastic bursting illustrated in Fig. 2. If just one delay feedback term is present, the bursting appears as described in Zheng and Pikovsky (2018). Because, after a pulse, a feedback force acts as a kick on the unit after delay time ${{\hat{\tau }}}$, there is an increased probability *p* for a next pulse to be induced. Thus, each spontaneously excited pulse can have a random number of “followers” separated by a time interval $\tau ={\hat{\tau }}+\tau _r$; together, they constitute a regular burst. Here, we take into account that the delay-induced effect is not instant, but rather it takes some relatively small response time $\tau _r$ for the unit to generate a spike after receiving a delayed kick, as described in Ref. Zheng and Pikovsky (2019). Generally speaking, the delay-induced spike occurs with some deviation around the effective time delay, i.e., not exactly at $t+{\hat{\tau }}+\tau _r$, but for simplicity, we assume the deviation can be ignored. The essential parameter of the stochastic bursting is the probability *p* to induce a follower, and we discussed in Ref. Zheng and Pikovsky (2018) the ways to calculate it.

In the case of two delayed feedback signals, after a spontaneous pulse at time *t*, there are two times $t+\tau _1$ and $t+\tau _2$ at which the followers can appear independently, with probabilities $p_1$ and $p_2$, and these probabilities are the same as for single time-delay feedbacks, as shown schematically in Fig. 2b. However, at the next level, there is an interaction of delayed kicks, which makes the problem essentially more difficult than that of one delayed feedback. Below in this paper we will assume that times $\tau _1$ and $\tau _2$ are incommensurate. Thus, we exclude resonances like $\tau _1=2\tau _2$, which will be treated in a separate work. We stress here that practically, because we consider relatively weak feedback, the number of spikes in a burst is not large. This means that “incommensurability” should be understood in a weak sense—as absence of resonances $m\tau _1=n\tau _2$ with small integers *m*, *n*.

If both followers at times $t+\tau _1$ and $t+\tau _2$ are present, at time $t+\tau _1+\tau _2$ there will be an overlap of two incoming feedback kicks. Generally, there are many such overlaps possible, at times $t+ k\tau _1+l\tau _2$ with *k* and *l* being both positive integers. (Here, due to incommensurability mentioned, for each overlap there is only one pair of the integers *k*, *l* which yields it.) Thus, another probability $p^{(2)}$ to induce a spike mediated by the overlap of two incoming kicks is needed. Generally, one must calculate this probability de novo. One may employ methods similar to those used for determining $p_1$ and $p_2$. However, when the two feedbacks are both weak and independent, we can assume a linear dependence of the probability to induce a follower on the incoming pulse amplitude, which makes an approximation $p^{(2)}\approx p_1+p_2$, to be adopted below, reasonable.

It is easy to see that potential followers of a spontaneous spike form a tree, as illustrated in Fig. 2b. On this tree, there can be non-branching paths, which correspond just to sequences of followers separated by time intervals $\tau _{1,2}$, which appear with probabilities $p_{1,2}$. However, any branching leads to an overlap, so we use $p^{(2)}$ to calculate the probability to observe the corresponding induced spike. We note here that the considered stochastic model is not a standard branching process, because of the overlaps.

It is instructive to start with the simplest overlap at time $\tau _1+\tau _2$. The probability to induce a spike at $t=\tau _1+\tau _2$ given a spike at $t=0$, i.e., ${\tilde{P}}(\tau _1+\tau _2)$, can be calculated as

5 $$\begin{aligned} \begin{aligned} {\tilde{P}}(\tau _1+\tau _2)&=(1-p_2)p_1p_2+(1-p_1)p_1p_2+p_1p_2p^{(2)}\\&\quad \approx 2p_1p_2. \end{aligned} \end{aligned}$$

**Fig. 3:**
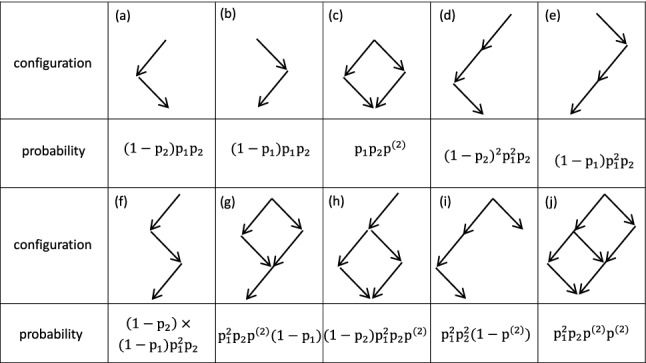
Configurations and the corresponding probability to induce a spike at the cross section $\tau _1+\tau _2$ (**a**)–(**c**) and $2\tau _1+\tau _2$ (**d**)–(**j**) of the lattice shown in Fig. 2b

Here, the first line in Eq. (5) describes contributions of different configurations with corresponding probabilities, as shown in Fig. 3a–c.

Similarly, we can calculate the probability to induce a spike at the time-delayed instant $2\tau _1+\tau _2$ as

6 $$\begin{aligned} \begin{aligned} {\tilde{P}}(2\tau _1+\tau _2)&=(1-p_1)p^2_1p_2+(1-p_1)(1-p_2)p^2_1p_2\\&\quad +(1-p_2)(1-p_2)p^2_1p_2+p^2_1p_2p^{(2)}p^{(2)}\\&\quad +(1-p_2)p^2_1p_2p^{(2)}+p_1p_2(1-p^{(2)})p_1p_2\\&\quad +p_1p_2p^{(2)}(1-p_1)p_1\\&\quad \approx 3p^2_1p_2. \end{aligned}\nonumber \\ \end{aligned}$$

The corresponding configurations and their probabilities are shown in Fig. 3d–j.

Now, by induction, it is easy to extend to the general case. One can easily check that the general expression

7 $$\begin{aligned} {\tilde{P}}(k\tau _1+l\tau _2)\approx \left( {\begin{array}{c}k+l\\ k\end{array}}\right) p^{k}_1p^{l}_2 \end{aligned}$$

is consistent with calculation of the induced probability ${\tilde{P}}(k\tau _1+l\tau _2)$ on the base of the “parent” probabilities ${\tilde{P}}((k-1)\tau _1+l\tau _2)$ and ${\tilde{P}}(k\tau _1+(l-1)\tau _2)$. By iteration of the relationship

8 $$\begin{aligned} \begin{aligned}&{\tilde{P}}(k\tau _1+l\tau _2)\\&\quad =(1-{\tilde{P}}(k\tau _1+(l-1)\tau _2)) {\tilde{P}}((k-1)\tau _1+l\tau _2)p_1\\&\qquad +(1-{\tilde{P}}((k-1)\tau _1+l\tau _2)){\tilde{P}}(k\tau _1+(l-1)\tau _2)p_2\\&\qquad + {\tilde{P}}((k-1)\tau _1+l\tau _2){\tilde{P}}(k\tau _1+(l-1)\tau _2)(p_1+p_2)\\&\quad ={\tilde{P}}((k-1)\tau _1+l\tau _2)p_1+{\tilde{P}}(k\tau _1+(l-1)\tau _2)p_2 \end{aligned} \end{aligned}$$

on the directed tree lattice, it is easy to obtain Eq. (7). We stress that expression (7) is valid only under assumption $p^{(2)}\approx p_1+p_2$; in a general case $p^{(2)}\ne p_1+p_2$, we could not derive a simple expression for these probabilities.

Having determined the probabilities of the followers, we now derive statistical properties of the bursts. To calculate the total firing rate $\mu$, we have to determine the average number of spikes in a burst. The probability to have *k* spikes with $\tau _1$ separation and *l* spikes with $\tau _2$ separation in the burst is the product of ${\tilde{P}}(k\tau _1+l\tau _2)$ and probability $1-p_1-p_2$, which is the probability to generate no spikes further. Thus, the total firing rate is

9 $$\begin{aligned} \begin{aligned} \mu&=\lambda (1-p_1-p_2)\sum \limits _{k, l}^{}{\tilde{P}}(k\tau _1+l\tau _2)(k+l)\\&\quad \approx \frac{\lambda }{1-p_1-p_2}\;. \end{aligned} \end{aligned}$$

**Fig. 4:**
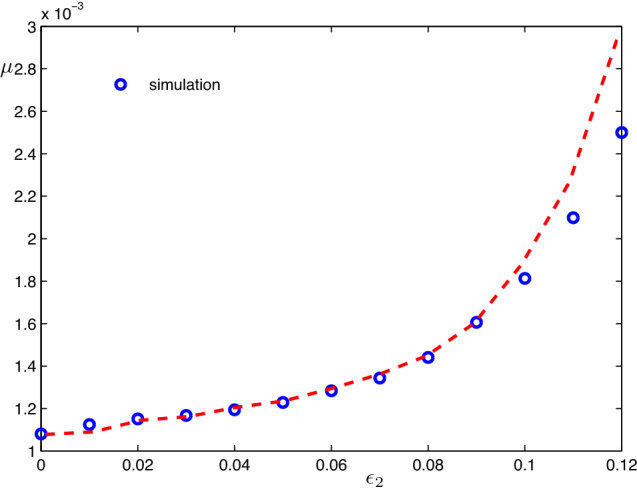
Spike rate of the single excitable unit with two delayed feedbacks, where $\epsilon _1$ is set to be 0.12 and $\epsilon _2$ is varied. The blue circles represent the result by direct simulation of Eq. (1), and the red dashed line represents the analytical result described by Eq. (9). Here, $\tau _1$ and $\tau _2$ are chosen as 500 and 600, respectively

We check this relation numerically in Fig. 4. Throughout the paper, the values of parameters for *a* and *D* are fixed as $a=0.95$ and $D=0.005$. This yields the spontaneous spike rate $\lambda =6.64\times 10^{-4}$, as calculated from Eq. (4). The probabilities to induce a spike can be calculated by virtue of solving a forced Fokker–Planck equation numerically as described in Ref. Zheng and Pikovsky (2018). For the delay coupling amplitudes $\epsilon =0.1$ and $\epsilon =0.12$, this gives values $p=0.25$ and $p=0.39$, respectively. Furthermore, in this case, the empirical value of the response time $\tau _r$ is approximately $\tau _r\approx 7$. In Fig. 4, we set the value of $\epsilon _1$ fixed, i.e., $\epsilon _1=0.12$ and thus $p_1=0.39$, and vary the strength of the second feedback $\epsilon _2$. As shown in Fig. 4, the analytic result described by Eq. (9) predicts well the spike rate when $\epsilon _2$ is small and moderate; deviations appear for large values of $\epsilon _2$.

Our next goal is to calculate correlations and spectra of stochastic bursting in this system. The autocorrelation function *C*(*s*) can be represented in terms of the joint probability to have a spike in the time interval $(t,t+{\varDelta }t)$ and a spike in the time interval $(t+s,t+s+{\varDelta }t)$:

10 $$\begin{aligned} C(s)=\frac{1}{T}\int _{0}^{T}\mathrm{d}t\lim \limits _{{\varDelta }t\rightarrow 0} \frac{P(t,t+{\varDelta }t; t+s,t+s+{\varDelta }t)}{{\varDelta }t^2}.\nonumber \\ \end{aligned}$$

Since the joint probability $P(t,t+{\varDelta }t; t+s,t+s+{\varDelta }t)$ is the product of the probability to fire a spike in the time interval $[t, t+{\varDelta }t]$, i.e., $\mu {\varDelta }t$ , and the conditional probability to fire a spike within the time interval $[t+s,t+s+{\varDelta }t]$ given a spike at time *t* (see also Ref. Zheng and Pikovsky 2019), i.e., $\sum _{k,l}{{\tilde{P}}}(k\tau _1+l\tau _2)\delta (s\pm (k\tau _1+l\tau _2)){\varDelta }t$, we obtain by using expression (7)

11 $$\begin{aligned} \begin{aligned} C(s)&=\mu \sum _{k,l}{{\tilde{P}}}(k\tau _1+l\tau _2)\delta (s\pm (k\tau _1+l\tau _2))\\&\approx \mu \sum _{k,l}\left( {\begin{array}{c}k+l\\ k\end{array}}\right) p^{k}_1p^{l}_2\delta (s\pm (k\tau _1+l\tau _2)). \end{aligned} \end{aligned}$$

The Fourier transform of the correlation function gives the power spectrum:

12 $$\begin{aligned} \begin{aligned} S(\omega )=&\int _{-\infty }^\infty \mathrm{d}s\;C(s)e^{-i\omega s}\\ \approx&\mu \sum _{k,l;k+l>0}\left( {\begin{array}{c}k+l\\ k\end{array}}\right) p^{k}_1p^{l}_2e^{ik\omega \tau _1+il\omega \tau _2}+\text {c.c.}+\mu \\ =&\mu \sum _{m=0}^\infty (p_1e^{i\omega \tau _1}+p_2e^{i\omega \tau _2})^m+\text {c.c.}-\mu =\\ =&2\text {Re}\left( \frac{\mu }{1-p_1e^{i\omega \tau _1}-p_2e^{i\omega \tau _2}}\right) -\mu . \end{aligned} \end{aligned}$$

**Fig. 5:**
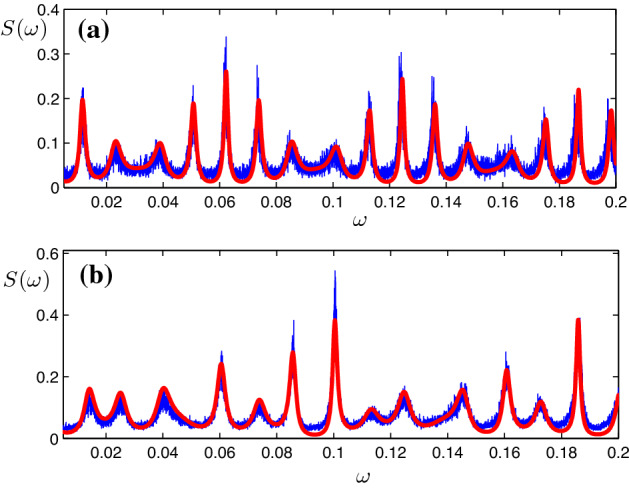
Power spectral density of a noisy excitable unit with **a** two-delay feedback and **b** three-delay feedback. For the direct simulation of Eq. (1) (blue lines), the parameters are chosen as $\epsilon _1=0.12, {\hat{\tau }}_1=500, \epsilon _2=0.1, {\hat{\tau }}_2=600$ in two-delay case and $\epsilon _1=\epsilon _2=\epsilon _3=0.1, {\hat{\tau }}_1=300, {\hat{\tau }}_2=430, {\hat{\tau }}_3=500$ for Eq. (13) in three-delay case. The red lines are the analytic results described by Eq. (12) for two-delay case and Eq. (13) for three-delay case. The power spectral density of the spike train is multiplied by the power spectral density of the shape function (color figure online)

We compare this theoretical prediction with the results of numerical simulation in Fig. 5a.

Using the same method, a noisy excitable unit with *m* delays described by the following Langevin equation

13 $$\begin{aligned} \begin{aligned} {\dot{\theta }}&=a+\cos \theta +\epsilon _1(a+\cos \theta (t-{\hat{\tau }}_1))+\cdots \\&\quad +\epsilon _m(a+\cos \theta (t-{\hat{\tau }}_m))+\xi (t) \end{aligned} \end{aligned}$$

can be studied. Here, the total spike rate can be expressed as $\mu =\lambda /(1-\sum \limits _{l=1}^{m}p_l)$, where $p_l$ is the probability to induce a spike by the delay feedback with strength $\epsilon _l$ and time delay ${\hat{\tau }}_l$. (We remind that we consider only the case of a weak feedback, where all $p_l$ are small.) The power spectral density of the corresponding spike train is described by the following formula:

14 $$\begin{aligned} S^{(m)}(\omega )\approx 2\text {Re}\left( \frac{\mu }{1-\sum \limits _{l=1}^{m}p_le^{i\omega \tau _l}}\right) -\mu , \end{aligned}$$

where $\tau _l={\hat{\tau }}_l+\tau _r$. Since the condition of timescale separation becomes harder to fulfill for large *m* (one needs the time intervals between pulses to be large), practically only the case $m=3$ is tested in Fig. 5b.

As shown in Fig. 5a, b, the analytic results described by Eq. (12) for two delayed feedbacks and Eq. (14) for three delayed feedbacks agree well with the direct simulation of the Langevin Eqs. (1) and (13), respectively.

**Fig. 6:**
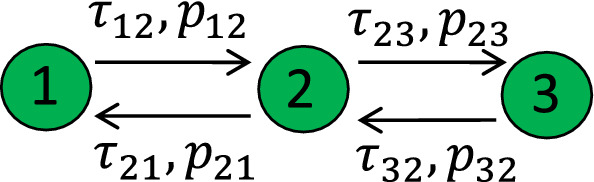
Schematic description of three delay-coupled noisy excitable units in a chain, where $p_{ij}$ is the probability to induce a spike due to delay feedback with strength $\epsilon _{ij}$ and time delay $\tau _{ij}$

## Delay coupling in a chain of three units

Here, we use the approach above to study a network of three delay-coupled noisy excitable units. The scheme of coupling is presented in Fig. 6, i.e., it is a star-type network with a central unit 2 (a hub) coupled to peripheral units 1 and 3. As is clear from Fig. 6, there are four delay times and four probabilities to induce a follower.

The Langevin equations, describing this network, read

15 $$\begin{aligned} \begin{aligned} {\dot{\theta }}_1&=a+\cos \theta _1+\epsilon _{21}(a+\cos \theta _2(t-{\hat{\tau }}_{21}))+\xi _1(t),\\ {\dot{\theta }}_2&=a+\cos \theta _2+\epsilon _{12}(a+\cos \theta _1(t-{\hat{\tau }}_{12}))\\&\quad +\epsilon _{32}(a+\cos \theta _3(t-{\hat{\tau }}_{32}))+\xi _2(t),\\ {\dot{\theta }}_3&=a+\cos \theta _3+\epsilon _{23}(a+\cos \theta _2(t-{\hat{\tau }}_{23}))+\xi _3(t). \end{aligned} \end{aligned}$$

Here, $\epsilon _{ij} (i,j=1,2,3)$ is the delay feedback strength from unit *i* to unit *j* with delay time ${\hat{\tau }}_{ij}$, and $\xi _i(t) (i=1,2,3)$ is the Gaussian white noise in unit *i* with $\langle \xi _i(t)\rangle =0$, $\langle \xi _i(t)\xi _j(t')\rangle =2D\delta _{ij}\delta (t-t')$. In the absence of delay feedback, i.e., $\epsilon _{ij}=0$, the three units fire spontaneously with constant rates $\lambda _i (i=1, 2, 3)$, described by Eq. (4). For simplicity, the noise intensities here in the three units are chosen to be the same. When the delayed feedback is included, i.e., $\epsilon _{ij}\ne 0$, each spontaneous spike in unit 1 will induce another spike in unit 2 with probability $p_{12}$ after time delay $\tau _{12}$, and the induced spike in unit 2 will generate spikes in unit 1 with probability $p_{21}$ after time delay $\tau _{21}$ and in unit 3 with probability $p_{23}$ after time delay $\tau _{23}$. Here, $\tau _{ij}$ includes the response time, i.e., $\tau _{ij}={\hat{\tau }}_{ij}+\tau _{r}$.

Thus, each spontaneous spike, which plays a role of leader, is followed by random number of induced spikes (followers) across the network. Noteworthy, the above description is based on timescale separation as described in Sect. 2. The relation to the model of one excitable unit with two feedbacks described in Section 2 is evident when one considers effective feedbacks from unit 2 to itself: There are two effective delay feedback channels, one with the probability $p_{21}p_{12}$ and time delay $\tau _{21}+\tau _{12}$ to induce a spike back into unit 2 itself through unit 1 and the other one through unit 3 with probability $p_{23}p_{32}$ and time delay $\tau _{23}+\tau _{32}$. Therefore, the total spike rate of unit 2 can be represented as

16 $$\begin{aligned} \mu _2=\frac{\lambda _2+p_{12}\lambda _1+p_{32}\lambda _3}{1-(p_{21}p_{12}+p_{23}p_{32})}. \end{aligned}$$

Here, the numerator $\lambda _2+p_{12}\lambda _1+p_{32}\lambda _3$ represents the total rate of first spikes in a burst in unit 2. These are spikes initiated spontaneously in unit 2 (rate $\lambda _2$), and the first followers in unit 2 of spikes spontaneously created in units 1 and 3 (rates $p_{12}\lambda _1$ and $p_{32}\lambda _3$, respectively). The denominator in (16) comes from the same summation as in Eq. (9).

Similarly, we can express the total spike rate $\mu _1$ of neuron 1 as

17 $$\begin{aligned} \begin{aligned} \mu _1&=\lambda _1+\mu _2p_{21}\\&=\lambda _1+\frac{\lambda _2+p_{12}\lambda _1+p_{32}\lambda _3}{1-(p_{21}p_{12}+p_{23}p_{32})}p_{21}, \end{aligned} \end{aligned}$$

since the leading spikes in unit 1 are either the spontaneous ones created in unit 1, or induced from total spikes of unit 2. For unit 3, the total rate is

18 $$\begin{aligned} \begin{aligned} \mu _3&=\lambda _3+\mu _2p_{23}\\&=\lambda _3+\frac{\lambda _2+p_{12}\lambda _1+p_{32}\lambda _3}{1-(p_{21}p_{12}+p_{23}p_{32})}p_{23}. \end{aligned} \end{aligned}$$

**Fig. 7:**
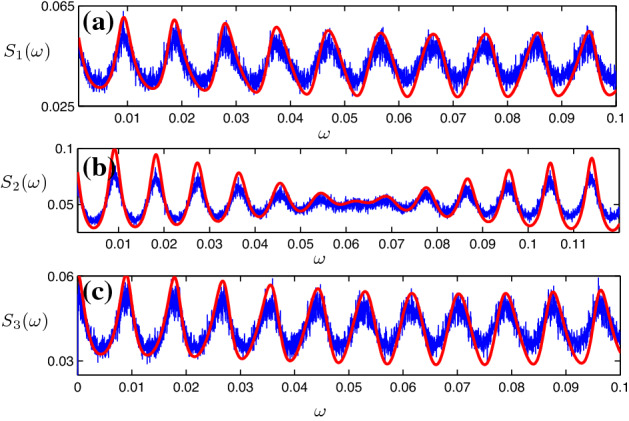
Power spectral density of neurons 1 (**a**), 2 (**b**) and 3 (**c**) in a chain of three delay-coupled noisy excitable neurons. Values of parameters are $\epsilon _1=\epsilon _2=\epsilon _3=0.12$, $\tau _{12}=350, \tau _{21}=300, \tau _{23}=300, \tau _{32}=400, a=0.95$ and $D=0.005$. Noteworthy, all the power densities are multiplied by the power spectral density of the shape function $S_H$, similar to Ref. Zheng and Pikovsky (2018, 2019). Blue lines: theory; red lines: analytic results (color figure online)

Using the same method as described in Sect. 2, we can write the power spectral density of a spike train in unit 2 as

19 $$\begin{aligned} S_2(\omega )\approx 2\text {Re}\left( \frac{\mu _2}{1-{\bar{p}}_1e^{i\omega {\bar{\tau }}_1}- {\bar{p}}_2e^{i\omega {\bar{\tau }}_2}}\right) -\mu _2, \end{aligned}$$

where ${\bar{p}}_1=p_{21}p_{12}$, ${\bar{\tau }}_1=\tau _{21}+\tau _{12}$, ${\bar{p}}_2=p_{23}p_{32}$ and ${\bar{\tau }}_2=\tau _{23}+\tau _{32}$. Similarly, taking into account all the delayed feedback loops connecting neuron 1, we obtain the power spectral density of neuron 1 as follows:

20 $$\begin{aligned} \begin{aligned} S_1(\omega )&\approx 2\text {Re}[\frac{\mu _1}{1-({\bar{p}}_1e^{i\omega {\bar{\tau }}_1} +{\bar{p}}_1{\bar{p}}_2e^{i\omega T}\sum \limits _{n>0}({\bar{p}}_2e^{i\omega {\bar{\tau }}_2})^n)}]\\&\quad -\mu _1\\&=2\mu _1\text {Re}\left( \frac{1-{\bar{p}}_2e^{i\omega {\bar{\tau }}_2}}{1-{\bar{p}}_{1} e^{i\omega {\bar{\tau }}_{1}}-{\bar{p}}_{2}e^{i\omega {\bar{\tau }}_{2}}}\right) -\mu _1. \end{aligned} \end{aligned}$$

Here, we denote $T={\bar{\tau }}_1+{\bar{\tau }}_2$ in the first line, and the term ${\bar{p}}_1{\bar{p}}_2e^{i\omega T}\sum \limits _{n>0}({\bar{p}}_2e^{i\omega {\bar{\tau }}_2})^n$ is due to the summation of all the feedback loops starting from neuron 1, connecting neuron 2 and 3 and then going back to neuron 1. By the same method, the power spectral density of neuron 3 is

21 $$\begin{aligned} S_3(\omega )\approx 2\mu _3\text {Re}\left( \frac{1-{\bar{p}}_1e^{i\omega {\bar{\tau }}_1}}{1-{\bar{p}}_{1} e^{i\omega {\bar{\tau }}_{1}}-{\bar{p}}_{2}e^{i\omega {\bar{\tau }}_{2}}}\right) -\mu _3. \end{aligned}$$

We compare these expressions with the results of direct numerical simulations of model (15) in Fig. 7a–c.

For networks of coupled units, it is instructive to calculate cross-correlations and cross-spectra. The cross-correlation between neuron 1 and 2 can be described in terms of the joint probability $P_{12}(t,t+{\varDelta }t; t+s,t+s+{\varDelta }t)$ that there are a spike in the time interval $(t+{\varDelta }t)$ in neuron 1 and a spike in the time interval $(t+s, t+s+{\varDelta }t)$ in neuron 2 (see also Ref. Zheng and Pikovsky 2019), i.e.,

22 $$\begin{aligned} \begin{aligned} C_{12}(s)&=\lim \limits _{{\varDelta }t\rightarrow 0}\frac{P_{12}(t,t+{\varDelta }t; t+s,t+s+{\varDelta }t)}{{\varDelta }t^2}\\&=\mu _1p_{12}\sum _{k,l}{\tilde{P}}(k{\bar{\tau }}_1+l{\bar{\tau }}_2)\delta (s-(k{\bar{\tau }}_1+l{\bar{\tau }}_2+\tau _{12}))\\&\quad +\mu _2p_{21}\sum _{k,l}{\tilde{P}}(k{\bar{\tau }}_1\\&\quad +l{\bar{\tau }}_2)\delta (s+(k{\bar{\tau }}_1+l{\bar{\tau }}_2+\tau _{21})). \end{aligned} \end{aligned}$$

Here, ${\tilde{P}}(k{\bar{\tau }}_1+l{\bar{\tau }}_2)$ is the probability to induce a spike at time $k{\bar{\tau }}_1+l{\bar{\tau }}_2$ after a spike, and according to Eq. (7), it is

23 $$\begin{aligned} {\tilde{P}}(k{\bar{\tau }}_1+l{\bar{\tau }}_2)\approx \left( {\begin{array}{c}k+l\\ k\end{array}}\right) {\bar{p}}^{k}_1{\bar{p}}^{l}_2. \end{aligned}$$

Substituting Eq. (23) into Eq. (22), we obtain the cross-correlation function

24 $$\begin{aligned} \begin{aligned} C_{12}(s)&\approx \mu _1p_{12}\sum _{k,l} \left( {\begin{array}{c}k+l\\ k\end{array}}\right) {\bar{p}}^{k}_1{\bar{p}}^{l}_2 \delta (s-(k{\bar{\tau }}_1+l{\bar{\tau }}_2+\tau _{12}))\\&\quad +\mu _2p_{21}\sum _{k,l}\left( {\begin{array}{c}k+l\\ k\end{array}}\right) {\bar{p}}^{k}_1{\bar{p}}^{l}_2\delta (s+(k{\bar{\tau }}_1\\&\quad +l{\bar{\tau }}_2+\tau _{21})). \end{aligned} \end{aligned}$$

The cross-spectral density between neurons 1 and 2 is just Fourier transform of the cross-correlation function, i.e.,

25 $$\begin{aligned} \begin{aligned} S_{12}(\omega )&=\int \limits _{-\infty }^{\infty }C_{ij}(s) e^{-i\omega s}\mathrm{d}s\\&\approx \frac{\mu _{1}p_{12}e^{-i\omega \tau _{12}}}{1-{\bar{p}}_1 e^{-i\omega {\bar{\tau }}_1}-{\bar{p}}_2e^{-i\omega {\bar{\tau }}_2}} \\&\quad + \frac{\mu _{2}p_{21}e^{i\omega \tau _{21}}}{1-{\bar{p}}_1 e^{i\omega {\bar{\tau }}_1}-{\bar{p}}_2e^{i\omega {\bar{\tau }}_2}}. \end{aligned} \end{aligned}$$

**Fig. 8:**
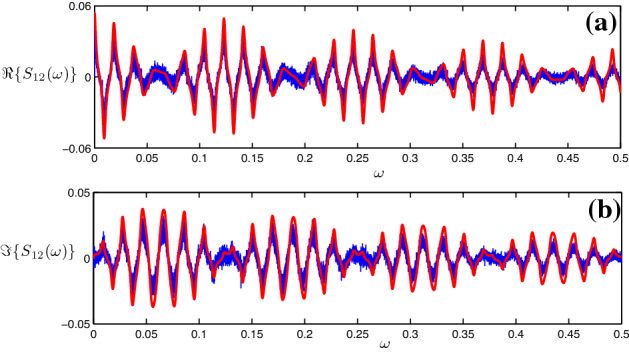
Real part (**a**) and imaginary part (**b**) of the cross-spectral density between neurons 1 and 2 in a chain of three delay-coupled noisy excitable neurons. Values of parameters are chosen the same as in Fig. 7. Noteworthy, the cross-spectral densities are also multiplied by the power spectral density of the shape function $S_H$ as in Fig. 7. Blue lines: theory; red lines: analytic results (color figure online)

As shown in Fig. 8 (see panels (a) and (b) for the real and the imaginary parts of the cross-spectral density), the analytic results agree well with direct simulation of Eq. (14).

## Conclusion

In summary, we investigated stochastic bursting in a single noisy excitable unit with multiple feedbacks and in a star-type network of three delay-coupled units. Both systems are the simplest examples of a neural network with an overlap of incoming spikes. In the deterministic case, this leads to polychronization (Izhikevich 2006a); in this sense, stochastic bursting in such a network may be referred to as noisy polychronization.

Our analysis is based on two approximations. One is that of timescale separation, valid for weak noise and large delay times. It allows us for modeling the process as a point one, so that only time instants of spikes are relevant for correlations and spectra. Another approximation is based on the assumption that the induced probabilities are small, and the probability for two overlapping inputs to induce a spike can be represented as a sum of the corresponding one-input probabilities. This latter assumption appeared to be extremely helpful, as it allowed us to express the probabilities of induced spikes in a simple closed form. We would like to point out that our analysis does not rely specifically on the theta model but is generally applicable to any excitable unit as long as the assumptions above (most important is that of separation of timescales) are met. Once the spontaneous firing rate and probabilities of generating of followers are known, the calculations of the total firing rate of each unit and of the pairwise correlations are straightforward.

As a result of our analysis, the spectra of the point stochastic bursting process have been analytically represented in a closed form. These power spectral and cross-spectral densities (12), (14) and (20–21) agree well with direct simulation of the original Langevin equations (1), (13) and (15). A general structure of the analytic expressions is the same: It yields a power spectrum with peaks at the delay times; the strengths of these peaks are roughly proportional to the corresponding probabilities to create a noise-induced follower. Remarkably, the cross-spectrum (25) has not a peak form, but rather looks like modulated oscillations, where characteristic modulation periods are the partial delays between the units.

Our approach works well for small numbers of interacting units and small numbers of feedback loops, because here the timescale separation is well justified. In a network with a large number of units and many connections, the spikes become denser, and the point process approximation might be violated. Similarly, for strong feedbacks the probability to induce a spike by overlapping inputs will be a non-trivial quantity (not a sum of one-input probabilities) as well.

At this point, we would like to mention several relevant studies of correlations in noisy neural networks. Pernice et al. (2011) investigated the pairwise correlation in networks of neurons with both excitatory and inhibitory interaction, based on the linear Hawkes processes. Trousdale et al. (2012) analyzed how the network structure influences the correlations based on the linear response theory (Lindner et al. 2005). There are also more sophisticated methods to investigate high-order correlations (Jovanović and Rotter 2016; Ocker et al. 2017). It would be interesting to consider the high-order statistics of stochastic bursting in the framework of the present model.

From the viewpoint of applications, we would like to mention that correlation functions of the spike trains are an important indicator of neural code in computational neuroscience (De La Rocha et al. 2007; Doiron et al. 2016; Nirenberg and Latham 2003). Our analytic results could be potentially compared with observed correlations and spectra, shedding light on the origin of observed correlations. In this context, it appears especially helpful that the basic expressions describing the spectral properties like (12), (14) and (25) contain only a few parameters. Therefore, experimentally observed spectra could be easily fitted to the derived analytic forms.
